# Measurement of cerebrovascular reserve by multimodal imaging for cerebral arterial occlusion or stenosis patients: protocol of a prospective, randomized, controlled clinical study

**DOI:** 10.1186/s13063-019-3967-2

**Published:** 2020-01-08

**Authors:** Zhi-peng Xiao, ke Jin, Jie-qing Wan, Yong Lin, Yao-hua Pan, Yi-chao Jin, Xiao-hua Zhang

**Affiliations:** Department of Neurosurgery, Renji Hospital, School of Medicine of Shanghai JiaoTong University, Shanghai, 200127 People’s Republic of China

**Keywords:** Stroke, Cerebrovascular reactivity, Cerebral blood flow, Multimodal image, Recurrence

## Abstract

**Background:**

Cerebrovascular reactivity (CVR) is the change in cerebral blood flow in response to a vaso-active stimulus, and may assist the treatment strategy of ischemic stroke. However, previous studies reported that a therapeutic strategy for stroke mainly depends on the degree of vascular stenosis with steady-state vascular parameters (e.g., cerebral blood flow and CVR). Hence, measurement of CVR by multimodal imaging techniques may improve the treatment of ischemic stroke.

**Methods/design:**

This is a prospective, randomized, controlled clinical trial that aimed to examine the capability of multimodal imaging techniques for the evaluation of CVR to improve treatment of patients with ischemic stroke. A total of 66 eligible patients will be recruited from Renji Hospital, Shanghai Jiaotong University School of Medicine. The patients will be categorized based on CVR into two subgroups as follows: CVR > 10% group and CVR < 10% group. The patients will be randomly assigned to medical management, percutaneous transluminal angioplasty and stenting, and intracranial and extra-cranial bypass groups in a 1:1:1 ratio. The primary endpoint is all adverse events and ipsilateral stroke recurrence at 6, 12, and 24 months after management. The secondary outcomes include the CVR, the National Institute of Health stroke scale and the Modified Rankin Scale at 6, 12, and 24 months.

**Discussion:**

Measurement of cerebrovascular reserve by multimodal image is recommended by most recent studies to guide the treatment of ischemic stroke, and thus its efficacy and evaluation accuracy need to be established in randomized controlled settings. This prospective, parallel, randomized, controlled registry study, together with other ongoing studies, should present more evidence for optimal individualized accurate treatment of ischemic stroke.

**Trial registration:**

Chinese Clinical Trial Registry, ID: ChiCTR-IOR-16009635; Registered on 16 October 2016.

All items are from the World Health Organization Trial Registration Data Set and registration in the Chinese Clinical Trial Registry: ChiCTR-IOR-16009635.

## Introduction

Stroke is the second most common cause of death and the major cause of disability worldwide after ischemic heart disease, especially in developing countries [1]. Ischemic stroke occurs when a blood vessel supplying blood to a part of the brain is obstructed, and it accounts for about 87% of all strokes. A remarkable risk of recurrent ischemic stroke was reported in patients with symptomatic, major cerebral arterial occlusion or stenosis [2]. Therefore, an effective therapeutic approach for intracranial arterial stenosis is urgently required.

The treatment of ischemic stroke has been investigated by a number of high-quality trials: The Japanese extracranial-intracranial bypass (EC-IC) bypass trial (JET2) study revealed that compared with the medical arm of the Japanese EC-IC bypass trial (JET) study including patients with cerebral blood flow (CBF) < 80% and cerebrovascular reactivity (CVR) < 10% as a historical control, the incidence of ipsilateral stroke recurrence was significantly lower in the JET2 study, demonstrating that EC-IC bypass surgery is unlikely to benefit patients with CBF > 80% or CVR > 10% [3]. Another recent trial involving aggressive medical treatment with or without stenting in high-risk patients with intracranial arterial stenosis (SAMMPRIS), demonstrated the use of aggressive medical management rather than percutaneous transluminal angioplasty and stenting (PTAS) with the Wingspan system in high-risk patients with atherosclerotic intracranial arterial stenosis [4]. AL Hasan conducted a trial for treating ischemic stroke, and showed a 14.7% risk of stroke or death in the stenting group versus 5.8% in the medical group at 30 days, and 23% in the stenting group versus 15% in the medical group at a median follow-up of 32.4 months. However, the treatment strategy of intracranial arterial stenosis or occlusion mainly depends on the degree of vascular stenosis, with or without consideration of hemodynamic factors at the distal end-to-side anastomosis of a bypass graft and CVR factors, or steady-state vascular parameters, such as CBF and cerebral blood volume (CBV). Hence, we present a study protocol for the measurement of CVR using multimodal imaging data for cerebral arterial occlusion or stenosis patients.

The CVR is the ability of cerebral vessels to dilate or constrict in response to challenges or maneuvers [5, 6]. In addition, CVR is thought to be an important index of the brain’s vascular health, and provides vascular-reserve information that is complementary to steady-state vascular parameters, including CBF and CBV [7, 8]. There have been two main approaches to measuring CVR. One approach attempts direct CBF measurements of the brain tissue with flow-sensitive imaging techniques such as positron-emission tomography (PET), nuclear medicine (NM) techniques, computed tomography (CT) perfusion, or magnetic resonance imaging (MRI) perfusion before and after a vasodilatory stimulus. The second approach involves transcranial Doppler (TCD) measurement of flow velocities (typically in the middle cerebral artery (MCA)) distal to a lesion both before and after a vasodilatory stimulus, with the increase flow velocity considered a surrogate for CVR [9–12]. We intend to precisely evaluate the change of CVR before and after surgical or medical treatments by multimodal image including MRI, CT, and single-photon-emission computed tomography (SPECT), so that we can make strategies for individualized accurate diagnosis and treatment for the ischemic stroke [3, 13].

Due to the lack of effective therapeutic approaches for intracranial arterial stenosis or occlusion, the present trial was registered at the Chinese Clinical Trial Registry database, and approved by the Center for Reproductive Medicine at Renji Hospital (Shanghai, China). The present trial was designed to determine whether multimodal imaging data can effectively enhance the treatment strategy for adult patients with intracranial arterial stenosis or occlusion.

## Methods/design

### Study design

**Fig. 1:**
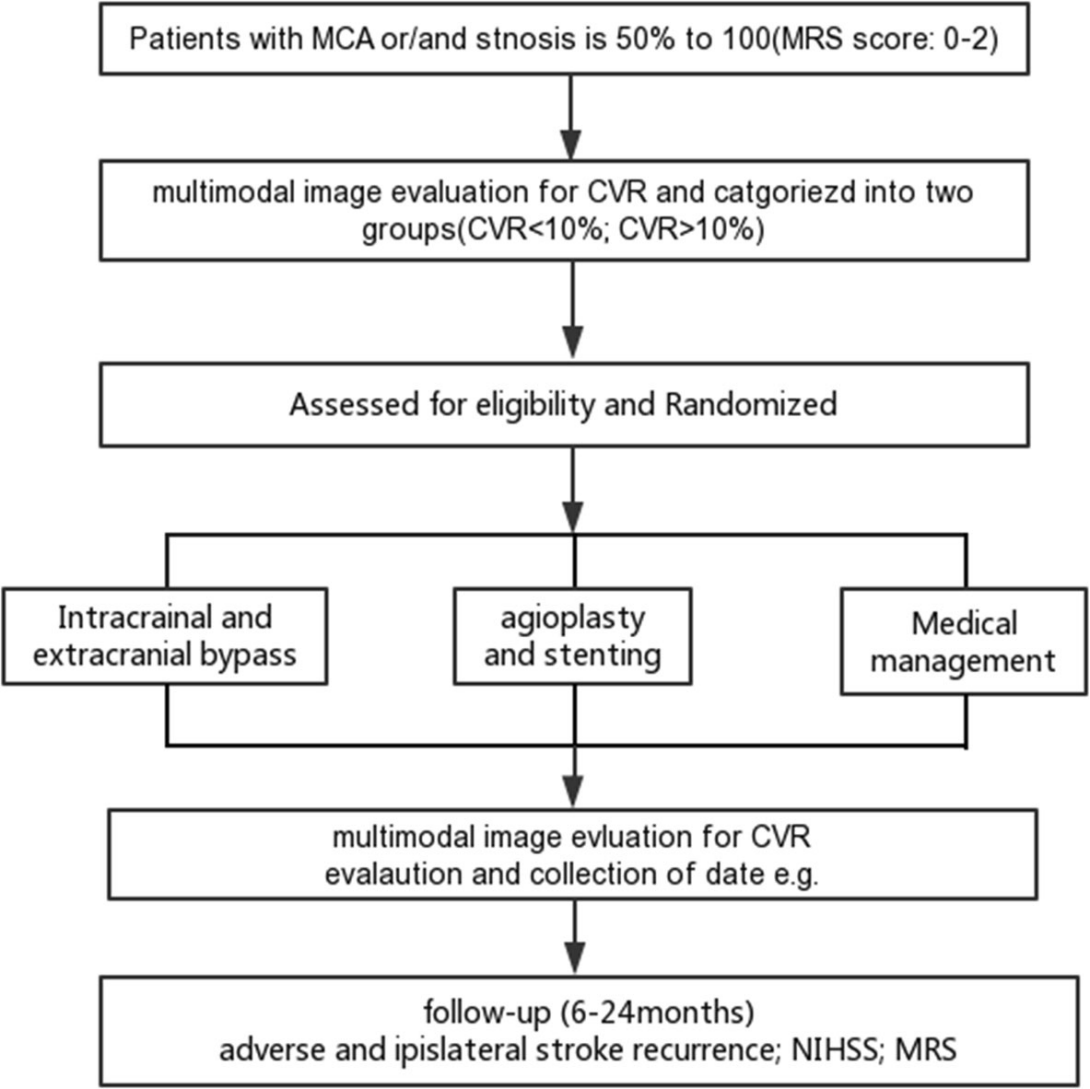
Study flowchart of our trial

This prospective, randomized, controlled clinical trial aimed to examine the efficacy of multimodal image data based on CVR to treat ischemic stroke. A total of 66 patients, who met the inclusion criteria, were admitted to Center for Reproductive Medicine at Renji Hospital, Shanghai Jiaotong University School of Medicine (Shanghai, China). The eligible patients were categorized based on CVR into two groups as follows: CVR > 10% group and CVR < 10% group. In addition, these two groups were randomly assigned to the groups of medical management, single angioplasty, PTAS, and IC-EC bypass in a 1:1:1 ratio. Fig. 1 shows the study flowchart of our trials.

### Inclusion and exclusion criteria

The eligible patients were identified if they met the following criteria: (1) clinical requirements: (a) men and women aged between 18 and 70 years, (b) independency in the activities of daily living (modified Rankin scale score of 0–2) on admission or after resuscitation; (2) radiological requirements: (a) occlusion or severe stenosis in the main trunk of the MCA or the supraclinoid segment of the internal carotid artery, (b) CT and MRI: no large infarction and no contrast enhancement in the infarcted area; and (3) signing the written informed consent form.

Exclusion criteria were as follow: (1) no independency in the activities of daily living (modified Rankin scale score of 3–5); (2) occlusive lesions of the cerebral arteries due to diseases other than atherosclerosis; (3) malignant tumors or multi-organ dysfunction involving the heart, liver, kidney, or lung; (4) myocardial infarction within the past 6 months; (5) uncontrolled diabetes showing a serum fasting blood glucose level of > 300 mg/dL, or requiring insulin; (6) hypertension with a diastolic blood pressure of > 110 mmHg;7; (7) artery-to-artery embolism; and (8) cardioembolism.

### Ethical approval and consent to participate

The study protocol was approved by the Ethics Committee of Renji Hospital. Furthermore, the present trial was registered at the Chinese Clinical Trial Registry (Registration No. ChiCTR-IOR-16009635). The trial was performed in accordance with the Declaration of Helsinki. All the participants signed the written informed consent form prior to start of study. The principal investigator explained the content of the research plan with the patient; including whether they agree to use of their data and asked for permission for the research team to share their relevant data with people from the universities taking part in the research or from the regulatory authorities. The principal investigator will obtain informed consent or assent from potential trial participants or the patient’s legal representative [14].

### Randomization and allocation concealment

In this trial, randomization sequence was generated by an independent institution that was not involved in the determination of eligibility.

### Treatment protocol

The eligible patients underwent multimodal imagine to measure CVR. Regional CBF was quantitatively measured more than 3 weeks after the last ischemic attacks using computed tomography perfusion (CTP) or SPECT (123I-IMP). A study of a small number of patients with chronic arterial stenosis compared ASL perfusion with ACZ challenge with iodine 123 *N*-isopropyl-piodoamphetamine (123I-IMP) SPECT, and the fixed concentration of CO_2_ was provided by a gas-delivery system using a pressure transmitter to control the gas blender with prospective gas-targeting algorithms. Subjects underwent SPECT/CTP scanning, and were asked to breathe normally for 10 min. The region of interest (ROI) was designated manually in the cerebral cortex in the territory of the ipsilateral MCA at the level of the anterior horn of the lateral ventricle. ROIs were also placed in the bilateral cerebellar hemispheres and in the contralateral MCA territory as reference. Regional CBF was expressed as relative values (%) to normal control values of each institute obtained from volunteers free of cerebrovascular disease. CVR was calculated as follows:

$$ CVR\ \left(\%\right)=\left[\left({CO}_2\  challenge\  CBF- rest\  CBF\right)/ rest\  CBF\right]\times 100. $$

All treatment procedures and specific operating protocols for the management of ischemic stroke in our trial were standardized based on current guidelines.

Surgical intervention was microsurgical end-to-side anastomosis of a superficial temporal artery branch to a cortical branch of the MCA. If the superficial temporal branch was felt to be inappropriate, the occipital artery could be used. For participants in the surgical group, preoperative and postoperative antithrombotic treatments were carried out by a neurosurgeon. Patients in the PTAS group received stenting when “alarm” symptoms were relieved after MT. Participants in the nonsurgical group continued to receive the antithrombotic treatment preferred by their physicians. Targets for controlling risk factor were 130/85 mmHg for blood pressure, 100 mg/dL for low-density lipoprotein, 150 mg/dL for triglycerides, and 7% for hemoglobin A1C.

### Study endpoints

Each patient was followed-up for 2 years by a neurologist and a neurosurgeon in each participating institute. Primary and secondary endpoints were defined as all adverse events and ipsilateral stroke recurrence at 6, 12, and 24 months after management, respectively. Neurological findings, intracranial CT/MRI, and CBF/CVR measurements were examined and reported at the time of enrollment and at 6 months, 1 year, and 2 years after enrollment. Evaluation of cognitive function and angiography were carried out at the time of enrollment and 2 years after enrollment. The functional outcomes were measured using CVR, the National Institute of Health stroke scale (NIHSS), and the Modified Rankin Scale (MRS) at 6, 12, and 24 months after the management.

### Data collection

The baseline data were collected including the following variables: hypertension, smoking status and whether diabetic, clinical presentation (i.e., initial ischemic stroke, on admission and before treatment); neurological functions (NIHSS and MRS), vascular stenosis, timing of management, treatment procedure, neurological conditions within 72 h after treatment, complications during hospitalization, follow-up, and presumed reasons of death. There is no storage of biological specimens for genetic or molecular analysis in the current trial and for future use in ancillary studies.

### Follow-up

In this trial, CTP and SPECT were followed-up 6 months after treatment. All the patients were followed-up after management by a neurosurgeon using a telephone interview or an in-person interview. The neurosurgeon was trained before the registry and was not involved in the treatment of ischemic-stroke patients. In outcomes after 6, 12, and 24 months, a MRS of 0–2 denoted a satisfactory outcome, and a score of 3–6 denoted a poor outcome.

Data verification was undertaken in 20% of all cases to assess the accuracy of data collection. The monthly audit, check of data quality, and statistical analysis were conducted by a third party who was in charge of notifying the principal investigator and Institutional Review Board of Renji Hospital about any issues that had arisen. Any serious adverse events were reported to the Institutional Review Board of Renji Hospital. Recommendations were forwarded to the principal investigators for reviewing risks and benefits. The Institutional Review Board had access to the interim results and made the final decision to terminate the trial.

### Sample size and data analysis

The number of patients included in the registry was equal to 60, and this trial involved 66 eligible ischemic-stroke patients, in which about 10% of patients were lost to follow-up. Data were presented as mean ± standard deviation (SD) for continuous variables, and as frequency for categorical variables. Significances between variables were analyzed using the chi-square test. Associations between clinical variables and outcomes were analyzed, and predictors of long-term outcome were identified using univariate and multivariate regression analyses. The difference was expressed as an odds ratio (OR, with 95% confidence interval (CI)), and *P* < 0.05 was considered statistically significant.

## Discussion

Previous studies have shown that medical management, PTAS, and IC-EC bypass can be applied to ischemic-stroke patients; however, which treatment method is more beneficial has remained elusive [4, 15]. The present trial was designed to indicate whether measurement of CVR using multimodal imaging is helpful to improve the treatment strategy for adult patients with intracranial arterial stenosis or occlusion.

Increased risk of stroke was found to be associated with hemodynamic failure, which can be assessed with measurement of CBF using (^15^O-)H_2_O PET [16]. This gold-standard technique, however, has not been presented for routine clinical imaging. Standardized blood oxygen-level-dependent functional MRI + CO_2_ is a noninvasive and potentially widely applicable method to assess whole-brain quantitative CVR. In addition, SPECT/CTP combined with CO_2_ challenge enables scholars to measure CBF and CVR, representing the degree of hemodynamic failure [17, 18].

It has been previously demonstrated that there is an association between CVR impairment and risk of stroke conserved across testing modality (TCD or nuclear medicine (NM) technique) as well as the nature of the vasodilatory stimulus (acetazolamide or variation in inspired CO_2_ levels). TCD is relatively inexpensive and widely available, while it does not provide additional information about brain parenchyma and is technically impossible in some cases due to lack of acoustic windows. In the present study, we accurately evaluated CVR for ischemic-stroke patients by multimodal imaging methods (MRI, CT, or SPECT), and explored the recurrence of ischemic stroke after treatment; thus, we can develop a new method for accurate diagnosis and treatment of ischemic stroke [9, 19, 20]. Our approach possesses a number of novel features compared with other relevant trials. Firstly, CVR in the ischemic-stroke patients was evaluated by multimodal imagine methods because those are widely applicable to assess whole-brain quantitative CVR. Some researchers have reported that SPECT is more sensitive than PET in the evaluation of CBF and cerebral perfusion, while its spatial resolution is negligible [21, 22]. The above-mentioned findings demonstrate that multimodal imaging techniques can accurately reflect changes in CVR. Secondly, the current study categorized the eligible patients into the groups of medical management, PTAS, and IC-EC bypass based on and the rates of CVR.

Finally, we recorded all the data related to the changes of CVR before and after treatment by multimodal imaging. The adverse events during the follow-up period were also taken and divided into ipsilateral stroke recurrence and all adverse events. We consequently found that such data could be help to analyze the effects of relevant confounding factors.

Our trial also indicated whether CVR can effectively and safely help improve the outcome in patients with intracranial arterial stenosis or occlusion. Regarding the major challenges in performing a clinical trial on ischemic stroke, the necessity for huge amounts of data is inevitable.

## Trial status

This protocol is the first version 1, which approved on 12 May 2019. The trial was started on 6 June 2018. We hope to achieve our research objectives by September 2020.

## Data Availability

Not applicable

## Abbreviations

CBV: Cerebral blood volume
CTP: Computed tomography perfusion
CVR: Cerebrovascular reserve
EC-IC: Extracranial-intracranial bypass
MCA: Middle cerebral artery
MRI: Magnetic resonance imaging
PET: Positron-emission tomography
PTAS: Percutaneous transluminal angioplasty and stenting
SPECT: Single-photon-emission computed tomography
